# Deaf Mutes

**Published:** 1902-06

**Authors:** E. A. Crockett

**Affiliations:** Boston, Mass.


### DEAF MUTES.[1]

BY E. A. CROCKETT, M.D., BOSTON, MASS.

I FEEL a little timid, gentlemen, in presenting an otological subject to a dental society, the more so because I think in the minds of a great many people the old impression of the curability of disease of the ear exists very much as it did thirty years ago. The stand-point of the average person at that time I think is very

[1] Address before the Harvard Odontological Society, November 21, 1901.

well illustrated by the story which is told at the Eye and Ear Infirmary, to the effect that one of the surgeons of the eye side goes over to a surgeon of the ear side, and says, "Doctor, I have been getting deaf for about a year, and I am really getting quite noticeably deaf now. I would like to have you look in my ear." And so the ear surgeon looks in the eye surgeon's ear, and the latter says, "Is it wax, doctor?" "No, it is not wax." "Well, that is all; good day."

I think there is no department of otology that illustrates this point more clearly than deaf mutism. There are about fifty thousand absolutely deaf individuals who have been educated at special schools for the education of the deaf, either by the lip method or by the finger method. That is, there are fifty thousand persons in the country who are absolutely unable to hear, and living in competition with persons of good hearing.

Some two years ago I became interested in the subject, and started to make a careful examination of the different mute schools in the State of Massachusetts. There are, in the different mute schools in this State, about three hundred pupils. There is the Horace Mann School in Boston, which is a day school, and the Clark Institute in Northampton, which is a boarding school; both are State institutions, although the Horace Mann is in the same general school system as the public schools of Boston.

I examined in the course of one winter about one hundred and thirty different mutes, largely with reference to the causation of the trouble, and I found that many of the one hundred and twenty-five or thirty had never been seen by an aurist before. That is, they had been sent directly to the institution for education by a special method,—in that school the oral method,—and denied the right of attendance at the public schools; and it had not been ascertained whether the deafness which they had was curable or incurable, or what the form of deafness was in each case. That was distinctly a hardship. Of course, we know there is a medical inspector to every public school in the city of Boston, and there was a medical inspector at that school, but he was merely a general physician and had no special knowledge of the ear, although every individual in the school, of course, needed treatment in that line. In the Clark Institute at Northampton, I think (although I have not yet examined those children), very much the same state of affairs exists.

In examining the children, I found, very much to my surprise, that there were eight or ten cases there that could be made to hear relatively perfectly well. I mean by that that with comparatively little treatment they could be made to attend the public schools, and would be able to hear, probably at the end of this room, what I am saying in this tone of voice. Fifty per cent. of the entire school were deaf from adenoids in the nasopharynx, which had either not been operated upon, or operated at too late a period to furnish relief. This fifty per cent., therefore, we may count as absolutely curable had they been treated at the proper time.

It is interesting to compare these statistics with the earlier statistics of deaf mutism in this country, and with the foreign statistics, particularly those of Norway and Sweden.

Fay has compiled a most elaborate book, and goes into a detailed account of some eight thousand cases of total deafness, and endeavors to prove that deaf mutism is largely an inherited condition. That is, he claims that twenty-five to thirty per cent. of the deaf mutes are cases of directly inherited deafness, and therefore form a special class in the community absolutely beyond help. And also his statistics prove that marriage of deaf persons, particularly marriages of deaf mutes, should not be allowed, or, at least, should be frowned upon by the profession. Among other things I think he claims that where two deaf mutes marry,—which is becoming very common in this country, as they are educated in coeducational schools, fifty per cent. of the children are deaf mutes also. That is a pretty large proportion. I think there have been about two thousand marriages of deaf mutes up to this time. Certainly all the European statistics seem to agree with Fay's standing upon this subject.

With that in view, I proceeded to examine all these cases in the Horace Mann School with great care,—first, as to whether they were hereditary or not; secondly, as to the exact amount of deafness present; and thirdly, as to whether they could be cured or not. As to the cause of the deafness, as I said, it is found that fifty per cent. come from adenoid disease, which is exceedingly common in the northern parts of this country and in northern countries all over the world. The fact that that is so large a factor accounts for the large amount of deafness in the northern portions of Europe, where the proportion of deaf mutes is exceedingly large.

Of the remaining fifty per cent., some ten or fifteen per cent. in this State were due to an epidemic of cerebrospinal meningitis, which we had some five or six years ago. At that time something like fifteen or twenty additional pupils came to the Horace Mann School. The deafness resulting from this disease will always be absolutely incurable, but never hereditary. A considerable proportion of the remainder came from the injuries of the ear-drum and middle ear from the discharging ears of scarlet fever and diphtheria in infancy, a condition curable had the process been treated in the beginning.

The remarkable part of the statistics to me was that out of the entire number of cases examined there were only two which could be considered to be hereditary, and those two were children of the same family, as they were twin sisters. In those two children no amount of investigation and questioning of the parents and examination of the children could give a cause for the deafness other than that it was presumably hereditary, as it was noticed at an early age, and the children had no disease in infancy which could account for it. So out of the total number of cases, only those two had inherited the affliction, and only those two could transmit it to their children, had they had any. This puts a totally different view upon the subject from the stand-point of statistics.

There are two other diseases particularly productive of total deafness in early life: one is inherited syphilis and the other bronchopneumonia in infancy.

It therefore becomes particularly important to notice the hearing of young children and to treat the affections of the auditory apparatus in young children as early as it can possibly be done. As a matter of fact, it is the experience of nearly every aurist that children are very rarely brought in for treatment of deafness before three or four years of age. The parents will notice that a baby has not begun to talk at the usual age, and by the time the child is two years old they begin to get a little bit worried about it; but they usually wait until the child is three before they come to see us. It is very important to determine as quickly as possible whether the failure to talk is from a mental lesion, from an ear lesion, or from delayed development. You will see children sometimes that at the age of two and one-half years will not talk, and after that age will talk fluently. It is exceedingly easy, if you have a chance to be with a child at all, to tell at a pretty early age whether they hear or not. If you only see the child once, it is pretty nearly impossible to tell accurately. If you are in the room with a child twenty or thirty minutes you can usually tell whether it hears or not, merely by watching its behavior to the ordinary sounds of the room, where if you try to test its hearing deliberately, and the child is strange to you, it will be absolutely impossible.

The particular thing you have to guard against, so far as my observation of the subject has gone, is the remarkable perception of vibration that all deaf patients have. For instance, it is interesting to see them instructing a totally deaf child in music, which they do by taking an ordinary piano and putting eight or ten children in a row with the fingers resting on the keys. The teacher will then run up the scale, and the children can feel the difference in the vibration of the different tones. So they can sing the scale, not having heard it at all. They simply receive the vibrations. It is a remarkably keen sense of vibration, developed to a very remarkable degree, and it makes it very difficult to test the hearing, for if you tap on the table, or slap your hands, or use any method of testing which has a perceptible vibration, the child will immediately show signs of hearing, where it does not hear at all. Consequently you should use sounds which, as nearly as possible, have no vibrations at all. The best test is the human voice.

It goes without saying that the only reason that nearly every child is mute is simply that it either has become deaf before the hearing period,—*i.e.*, before one or two years of age,—or else it has acquired deafness between the ages of two and five, and has forgotten what words it had already learned. Total deafness is almost unheard of. Of the entire one hundred and thirty children that I examined, there were only two or three absolutely deaf; nearly all could hear certain tones of the human voice, or some portion of the musical scale, usually in the upper portion. That is, some portion of the auditory nerve remained, and that is a very important point in the education of the child. If you can once give them an idea of what a tone really sounds like, you can get them to place the tone and use the voice better. It is of the greatest importance in the young child to define the exact amount of hearing and to bring it as quickly as possible to its best point of hearing.

There is one very interesting point about these children, and that is that they learn lip reading almost instinctively. I found three children who had been two or three years in the public schools and had advanced with the rest of their class, without the deafness being noticed at all. That is, they were natural lip readers, and as long as they could be with children and see them, they could tell what they said. It was noticed that at one time the children would respond perfectly, and the next minute did not respond at all, absolutely making no sign of recognition. At that time the children were unable to see the lips. You must be very careful about this point in testing young children, or you will be deceived once in a while in spite of yourself.

The children are nearly all of them exceedingly bright, and there is absolutely no trouble, by the time they are four or five years old, in distinguishing between idiocy and deafness. They learn difficult matters of education very much easier than persons of normal hearing do. It is important for these children to have their deafness recognized early, as I have said, and to be sent to school at the usual school period if possible. That sounds like a truism, but the majority of the pupils are not sent to the school until they are eight or nine years old. Of course, it is difficult for them to begin with the kindergarten then and learn their A, B, Cs from the bottom up. At fifteen years of age, and older, it is difficult to teach them any method of communicating with the outside world except by the finger method, which of course is very readily learned, but is very awkward, as they cannot communicate except with people who know the method. Where the child begins early, from three to five years old, it is not allowed to use the finger method at all, but is obliged to learn the oral method, reading with the lips. The teacher begins with simple words, and puts the child through the regular school course. So if a bright child begins at four years old, by the time it is fifteen it will be able to enter high school not over one or two years behind. Adults find it almost impossible to do anything at all with the lip method. The younger children, so the teachers tell me, if they possess too much hearing, will not pay much attention to the lip method, endeavoring to use the hearing and not the lips.

I think about a thousand pupils have been graduated from the schools of Massachusetts in the last fifteen or twenty years with the oral method, and the great majority of them have been well able to earn their own living and to pursue useful occupations, in many cases earning good living wages. That was a thing which was absolutely impossible twenty-five years ago.

But the most important point which I desire to make in the brief talk to-night is that if all the children who are deaf had had their deafness recognized and were treated intelligently at an early period, there would be at least from fifty to seventy-five per cent. less deaf mutes in the United States than there are at the present time; instead of being fifty thousand, there certainly would not be over twenty thousand, and probably not that many, because in this city the cerebrospinal epidemic has made a large number of deaf children which we would not find in other cities. This is a very important point, because anybody who has anything to do with children will find among those of his acquaintance a very considerable number who are thought to be peculiar or obstinate or stupid, or something of that sort, but a careful investigation will show a physical cause for it in their ears. So far as my experience with school children goes,—and in the last two years I have examined a large number,—I think it is not too much to state that practically no child is either obstinate or stupid without a physical cause for it. That is, they may not necessarily be deaf, but they are near-sighted, or possess some physical disease; there is some actual lesion to account for it. It is the natural habit of the growing child of from five to eight years old to be bright and happy and to be observant, provided he has not a physical lesion keeping him back. And if that physical lesion is corrected, the child is able to assume a position in after life that he is not able to take otherwise.

I found two or three very unusual cases; for instance, I found two cases of cretinism, and two idiots, and there were one or two other very unusual cases in the same class with the others, entirely without any justification at all.

Another question which comes into the subject of deaf mutism in education is the question of their marriage. All the coeducational schools in this State keep them in the school, or under observation, up to at least eighteen years of age. Of course, it is decidedly an open question whether this is wise or not, because they feel a good deal of sympathy with one another, and naturally form marriages with their associates. The statistics on that point are very interesting indeed. In the census records of the country up to 1850 there is only one case of marriage of deaf mutes, and it rises until it is something over a thousand cases in the last census report. But if we are to believe the modern statistics rather than the old statistics, we are unable to see that the children of those marriages need to be deaf, or stand any greater chance of being deaf than the children of ordinary marriages. The tendency to adenoid diseases is certainly inherited, but at the same time the operation which has come up in the last twenty years makes the disease perfectly curable, and consequently eliminates all that class of cases. All the cases which come from diseases like scarlet fever, measles, etc., are met by modern treatment, and are perfectly cured. Consequently the proportion of the deaf from the deaf marriages is so small that, while we may think it is or is not a good thing so far as the deaf people themselves go, it apparently has no importance so far as their children are concerned.

That question also concerns the people who are partially deaf. I was approached this year in regard to whether it was wise for two first cousins to marry, in whose family a certain amount of deafness existed. Careful analyses of the adult cases in that family showed that all the cases of deafness were of that class which would now be curable. The adult members of the family were sixty-five or seventy years old, or thereabouts, and at the time their diseases were contracted curative treatment was impossible. A careful analysis of their cases enabled it to be said that the younger members of the family could marry with absolute safety, so far as any deafness in their offspring might result; and it was, of course, a very important point to them.

There is no question, I think, so far as that goes, that there is practically only one disease of the ear which is hereditary, either directly or as a tendency, and that is the class of diseases which involve the auditory nerve itself, and the scapulo-vestibular region, which usually come on in adult life. That is, we can say, as a fairly general rule, that if the person has begun to be deaf early, the deafness is probably remediable, and it is probably not to be inherited in children born in after years.

That is about all that I desire to say, Mr. President, on the subject. I trust that it interests you as dentists. It interested me very much at the time I was making the investigations, particularly to find out how much change there had been in thirty years in etiology, and how different the prognosis of these apparently hopeless cases was.

